# Heat acclimation and thirst in rats

**DOI:** 10.14814/phy2.12642

**Published:** 2015-12-23

**Authors:** Christopher C. Barney, Elizabeth M. Schanhals, Justin L. Grobe, Bradley T. Andresen, Michael Traver

**Affiliations:** ^1^ Department of BiologyHope CollegeHollandMichigan

**Keywords:** Angiotensin II, intracellular thirst, kidney, rehydration, thermal dehydration, volemic thirst

## Abstract

The effects of heat acclimation on water intake and urine output responses to thermal dehydration and other thirst stimuli were studied in male Sprague–Dawley rats. Rats were heat acclimated by continuous exposure to a 34°C environment for at least 6 weeks. Thermal dehydration‐induced thirst was brought about by exposing the heat‐acclimated rats and control rats housed at 24°C to a 37.5°C environment for 4 h without access to food or water. Heat acclimation reduced evaporative and urinary water losses and the increases in plasma sodium and osmolality during thermal dehydration, which led to a reduction in thermal dehydration‐induced thirst. Heat acclimation reduced the rate of rehydration following thermal dehydration but did not alter the final rehydration level, indicating that heat acclimation does not alter the primary control of thermal dehydration‐induced thirst. Heat acclimation did not alter water intake or urine output following administration of hypertonic saline, which selectively stimulates intracellular thirst, but led to greater water intake following administration of angiotensin II, which plays an important role in extracellular/volemic thirst, and following water deprivation, which activates both thirst pathways. Cardiovascular responses to angiotensin II were not altered by heat acclimation. Heat acclimation thus reduces water loss during heat exposure in rats, but does not have major effects on thermal dehydration‐induced or extracellular thirst but does appear to alter volemic thirst.

## Introduction

Thirst and water intake are important physiological/behavioral mechanisms to prevent or restore water deficits and thus prevent or reduce the deleterious effects of dehydration. Although a variety of circumstances can cause dehydration and thirst, heat exposure might be one of the most important. During heat exposure humans and many other mammals increase evaporative water loss in order to keep core temperature from increasing to dangerous levels. If water is not available for drinking, this increased loss of water leads to thermal dehydration, which increases the risk of heat illness and heat stroke. As heat waves are associated with increased mortality (Poumadère et al. 2005; Wilson et al. 2013) and there are projections of increased frequency of heat waves and heat‐related mortality as global warming continues (Greene et al. 2011; Huang et al. 2011), it is important that we have a good understanding of physiological and behavioral responses to both short‐term and long‐term heat exposure, including the role of thirst in mitigating the effects of thermal dehydration.

In the rat, an animal widely used for studies on thirst, exposure to the heat increases the production and spreading of saliva as a means of evaporative cooling (Hainsworth 1967, 1968; Horowitz et al. 1983; Yanase et al. 1991). The increased salivary water loss leads to thermal dehydration and an increase in water intake when water is made available (Hainsworth et al. 1968; Nose et al. 1985, 1986; Barney and West 1990). The thirst induced by thermal dehydration is primarily intracellular in nature (Nose et al. 1985, 1986; Barney and West 1990) as plasma volume is defended during the initial stages of thermal dehydration (Horowitz and Samueloff 1979; Durkot et al. 1986; Barney and West 1990) and an intravenous preload of water, but not isotonic saline, significantly reduces thermal dehydration‐induced thirst (Barney 1997).

Thermal dehydration‐induced thirst in rats can be modulated by physiological or pathophysiological states. Water intake following heat exposure was increased in the dark phase of the light cycle compared to the light phase (Barney et al. 1995). In another experiment water intake was increased in spontaneously hypertensive rats compared to their normotensive controls (Barney et al. 1999). Old rats exhibited a reduced thirst response to thermal dehydration (Whyte et al. 2004). In this study, we were interested in determining the effects of heat acclimation on thermal dehydration‐induced thirst. Because heat acclimation has been shown to reduce heat‐induced evaporative water losses in rats (Horowitz et al. 1983; Gwosdow et al. 1985; Matthew 1997) and to reduce glomerular filtration rate and urine output (Chayoth et al. 1984), we hypothesized that heat acclimation would alter thermal dehydration‐induced thirst and salt and water excretion during thermal dehydration. In addition, we hypothesized that heat acclimation would alter thirst induced by water deprivation and by independent activation of intracellular and extracellular/volemic thirst pathways (Stricker and Sved 2000; McKinley et al. 2004).

## Materials and Methods

### Animals

Male Sprague–Dawley strain rats purchased from Harlan Laboratories (Indianapolis, IN) with initial weights from 190 to 240 g (7–8 weeks of age) were used for these experiments. Mean weight of the heat‐acclimated group (range 290–375 g) at the start of each experiment was significantly (*P* < 0.001) less than the mean weight of the control group (range 361–475 g). Rats were housed singly in hanging stainless steel cages. Upon arrival the rats were housed in an animal room kept at 24 ± 2°C for at least 1 week. Control rats remained in the animal room and the rats to be heat‐acclimated were transferred to a walk‐in environmental chamber kept at 31 ± 0.5°C. The temperature of the environmental chamber was increased by 0.5°C each day for 6 days such that the final acclimation temperature was 34 ± 0.5°C, in order to reduce the initial stress of heat acclimation (Gwosdow et al. 1985). The animal room and environmental chamber were illuminated from 7:00 am until 7:00 pm The rats had access to water and Purina rat chow ad lib except during the experimental periods. All experiments began between 8:00 and 10:00 am. Rats were allowed to acclimate to the heat for at least 6 weeks. When rats were used for more than one type of experiment, at least 2 weeks were allowed between experiments, with the rats remaining at their respective housing temperatures. Prior to the beginning of each experiment, the control and heat‐acclimated rats were allowed to adjust to a 25°C environment with water but not food for 1 h and then the rats were weighed. The experiments were approved by the Hope College Animal Care and Use Committee and followed Hope College's animal care and use guidelines and the *NIH Guide for the Care and Use of Laboratory Animals*.

### Thermal dehydration experiments

In the first experiment, 18 control rats and 18 heat‐acclimated rats were placed in modified (Barney and West 1990) Nalgene metabolism cages without access to food or water. Half of the rats in each group were placed in a walk‐in environmental chamber kept at 25 ± 0.5°C and the other half were placed in a walk‐in environmental chamber kept at 37.5 ± 0.5°C. After 4 h, the cages were removed from the chambers and the rats were reweighed. One heat‐acclimated rat unexpectedly died soon after removal from the 37.5°C chamber. The rats were then placed in other, standard Nalgene metabolism cages in the 25°C chamber and provided water but not food. Water intake and urine output were measured at 0.5, 1, 2, 3, and 4 h. Evaporative water loss during the exposure period was estimated by subtracting urine and fecal losses from the change in body weight over the 4‐h period. Percent rehydration (Barney and West 1990) was determined by dividing the water intake during the drink period by the exposure period evaporative and urine water losses plus the urine loss during the postexposure time when the rats had access to water (drink period) and then multiplying by 100%. Urine samples from both the exposure and drink periods were frozen at −80°C for later determination of sodium and potassium concentration by flame photometry using a Varian AA‐475 atomic absorption spectrophotometer.

In the second experiment, 18 control rats and 18 heat‐acclimated rats were divided into two groups each and exposed to 25 and 37.5°C, as in the first experiment. Fifteen minutes after the end the exposure period, each rat was anesthetized with methoxyflurane and a 3 mL blood sample was taken using cardiac puncture and a syringe containing 200 units of lithium heparin. Triplicate measurements of plasma sodium, potassium and protein concentrations, plasma osmolality, and hematocrit were determined as previously described (Barney et al. 1999). Due to technical problems, a blood sample was not obtained from one control and one heat‐acclimated rat exposed to 37.5°C.

### Thirst challenge experiments

Water deprivation – Twelve control and 12 heat‐acclimated rats were used to determine if responses to water deprivation were affected by heat acclimation. In this experiment, the rats were weighed and placed in Nalgene metabolism cages at 25°C with access to water but not food at the beginning of the adjustment period so water intake and urine output could be determined. Water intake and urine output were measured for 1 h. Then water was removed and powdered Purina rat chow was provided. After 24 h of water deprivation, the rats were reweighed and urine output, food intake, and feces output were determined. Water, but not food, was then provided and water intake and urine output were determined every hour for 4 h.

Hypertonic saline – Six control and six heat‐acclimated rats were lightly anesthetized with methoxyflurane and then administered intraperitoneal injections of 37°C hypertonic saline (0.75 mol/L NaCl, 10 mL/kg). Rats were then placed in Nalgene metabolism cages at 25°C with access to water but not food. At 1, 2, and 3 h, water intake and urine output were determined. After 3 h, urine was saved and frozen at −80°C. Sodium and potassium concentrations of the urine samples were determined as in experiment 1, and excretion rates were determined by multiplying the concentrations by the 3‐h cumulative urine output for each rat.

Intraperitoneal angiotensin II – As angiotensin II is a primary mediatory of volemic thirst, 12control rats and 11 heat‐acclimated rats were administered intraperitoneal injections of 200 *μ*g angiotensin II (Phoenix Pharmaceuticals, Inc., Mountain View, CA)/kg at 1 mL/kg to determine if heat acclimation alters this thirst pathway. Rats were then placed in Nalgene metabolism cages at 25°C with access to water but not food. At 1, 2 and 3 h, water intake and urine output were determined.

Intravenous angiotensin II – The next experiment was performed to determine if differences in the drinking response to intraperitoneal administration of angiotensin II between the control and heat‐acclimated rats might have been due to altered absorption of the angiotensin II into the blood. Eight control rats and eight heat‐acclimated rats were fitted with chronic jugular cannulas 2 days before infusion. The rats were anesthetized with 1 mg/kg acepromazine maleate, 100 mg/kg ketamine hydrochloride, and 20 mg/kg pentobarbital sodium injected intraperitoneally. The right jugular vein was cannulated with PE‐50 tubing, which was tunneled under the skin and exteriorized through a small incision at the back of the neck. The cannula was filled with heparinized (100 units/mL) saline and occluded with a stainless steel plug. Following recovery from the anesthetic the rats were returned to their home cages. For the infusion, the cannula of each rat was connected to a Sage infusion pump and 0.9% saline was infused at a rate of 0.0326 mL/min for 30 min. Then angiotensin II was infused at the rate of 0.25 *μ*g/kg‐min at the same flow rate for 90 min. Cumulative water intake and urine output were determined at the end of the saline infusion period and every 30 min during the angiotensin II infusion. Because of a procedural error, urine output was not determined in three rats from each group.

### Cardiovascular responses to angiotensin II

Eight control and eight heat‐acclimated rats were anesthetized as in experiment 6 with additional pentobarbital sodium being administered as needed throughout the experiment to maintain anesthesia. Colonic temperature was monitored and maintained between 36 and 37°C using a heating pad placed beneath each rat. The right jugular vein and the left carotid artery were cannulated with PE‐50 tubing filled with heparinized (100 units/mL) saline. Heparinized saline or angiotensin solution was infused into the jugular vein with a Sage infusion pump at the rate of 0.103 mL/min. Blood pressure was determined using a P‐1000B pressure transducer and a Narco‐Biosystems physiograph. Mean blood pressure was calculated by adding the systolic pressure to two times the diastolic pressure and dividing by 3. Following the cannulation procedures the rats were allowed to recover for 15 min and were then infused with angiotensin II (Sigma, St. Louis, MO) at doses of 0, 10, 30, and 90 ng/kg‐min for 5 min followed by 15‐min recovery periods. The blood pressure and heart rate during the last minute of the 5‐min infusion period were determined. The rats remained anesthetized throughout the experiment and were euthanized with an overdose of pentobarbital sodium administered through the venous cannula at the end of the experiment.

### Statistical analysis

The software package SYSTAT 10 was used for statistical analysis. The data are expressed as means ±** **1 SE. *T*‐tests, one‐way analysis of variance (ANOVA), two‐way ANOVA, two‐way ANOVA with repeated measures, and three‐way ANOVA with repeated measures were used for inferential statistics with significance set at the 95% confidence level.

## Results

### Body weight

**Figure 1:**
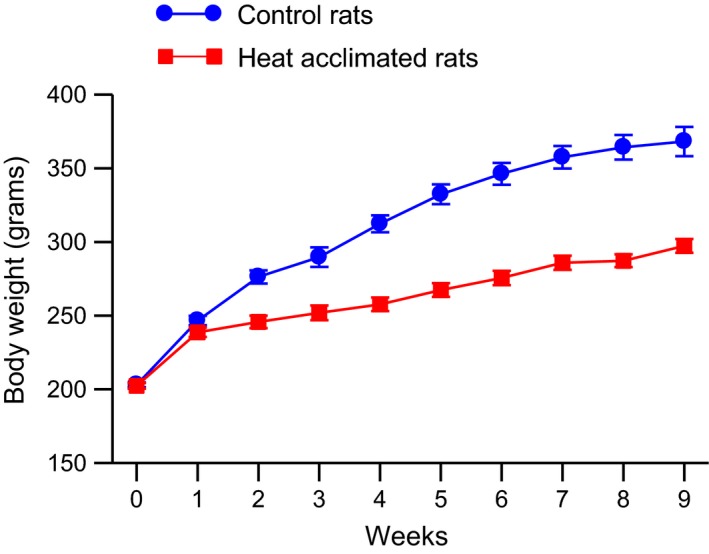
Mean ± SE body weight of one set of control and heat‐acclimated rats for 9 weeks of exposure. Control rats were kept at 24 ± 2°C and the heat‐acclimated rats were initially placed in an environmental chamber at 31 ± 0.5°C. The temperature of the environmental chamber was increased by 0.5°C each day for 6 days and then the temperature was kept at 34 ± 0.5°C for the remainder of the experiment. *N* = 15 for both groups. ANOVA indicated significant effects of group and time and a significant interaction between group and time on body weight.

Rats undergoing heat acclimation grew more slowly than did the control rats (Fig. 1, example data from one set of rats). One‐way ANOVA with repeated measures showed significant main effects of group (*F*_1,28_ = 61.55, *P* < 0.0001) and time (*F*_19, 28_ = 260.03, *P* < 0.0001) and a significant interaction between group and time (*F*_9,28_ = 29.32, *P* < 0.0001).

### Effects of heat acclimation and thermal dehydration on water intake and urine output

**Figure 2:**
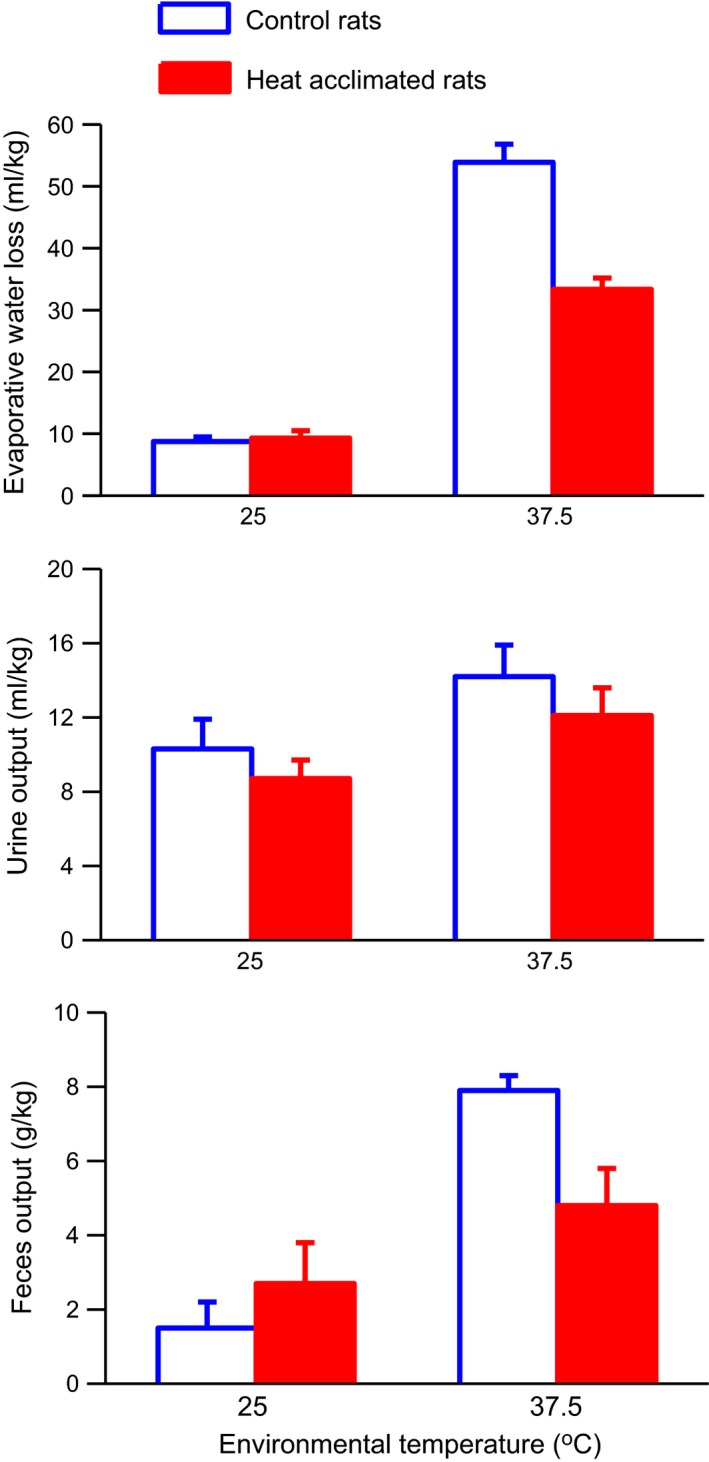
Mean evaporative water loss (*top*), urine output (middle) and feces output (*bottom*) in control and heat‐acclimated rats for 4 h of exposure to either a 25°C or 37.5°C environment. Error bars show SE. *n *=* *8 for the heat acclimated 37.5°C group and 9 for all other groups. ANOVA indicated significant effects of group and temperature and a significant interaction between group and temperature on evaporative water loss. ANOVA indicated a significant effect of temperature on urine output and feces output and a significant interaction between group and temperature on feces output.

Control and heat‐acclimated rats had similar evaporative water losses during the exposure period at 25°C (Fig. 2, top) as estimated by body weight changes corrected for fecal and urinary weight losses. Exposure to 37.5°C for 4 h increased evaporative water loss 6.2 times in the control rats and 3.6 times in the heat‐acclimated rats. Two‐way ANOVA of the evaporative water loss data showed significant main effects of group (*F*_1,31_ = 28.43, *P* < 0.0001) and temperature (*F*_1,31_ = 341.92, *P* < 0.0001) and a significant interaction between group and temperature (*F*_1,31_ = 31.75, *P* < 0.0001). Urine output (Fig. 2, middle) was slightly lower in the heat‐acclimated rats at both temperatures. Heat exposure caused an increase in urine output in both groups. Two‐way ANOVA of the urine output data showed a significant main effect of temperature (*F*_1,31_ = 6.30, *P* < 0.02), but no significant effect of group nor significant interaction between group and temperature. Heat exposure also caused an increase in feces output in both groups (Fig. 2, bottom) with the heat‐acclimated rats having a smaller increase than the control rats. Two‐way ANOVA of the feces output data showed a significant (*F*_1,31_ = 26.15, *P* < 0.001) effect of temperature and a significant interaction (*F*_1,31_ = 6.47, *P* < 0.02) between group and temperature on feces output.

**Figure 3:**
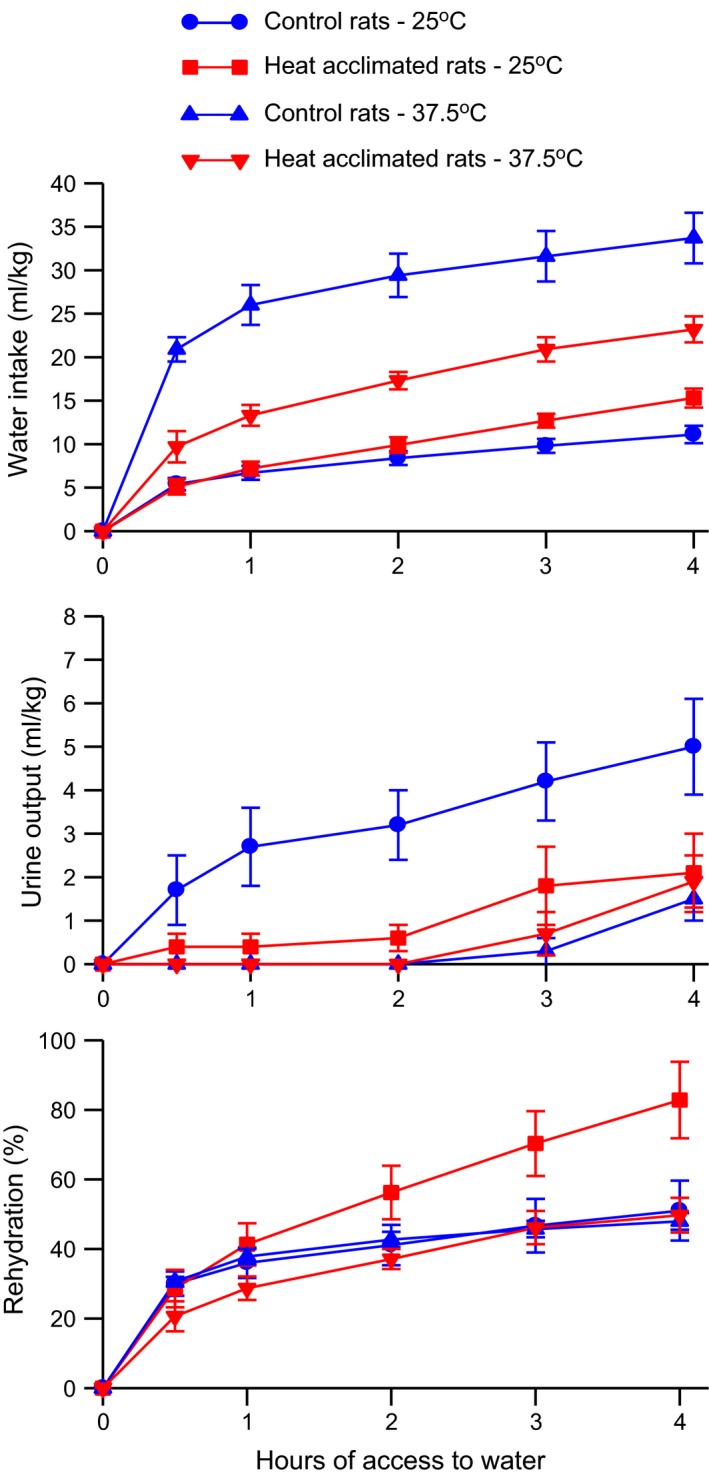
Mean ± SE cumulative water intake (*top*), urine output (*middle*), and percent rehydration (*bottom*) of control and heat‐acclimated rats for 4 h of access to water at an environmental temperature of 25°C following 4 h of exposure to either a 25°C or 37.5°C environment. *n *=* *8 for the heat acclimated 37.5°C group and 9 for all other groups. ANOVA indicated significant effects of group, temperature, and time and significant interactions between group and time, group and temperature, and temperature and time on water intake. ANOVA indicated significant effects of group, temperature, and time and a significant interaction between group and temperature on urine output. ANOVA indicated a significant effect of time and significant interactions between group and time and temperature and time on percent rehydration.

Water intake, urine output, and percent rehydration during the drink period are shown in Figure 2. Initially, control and heat‐acclimated rats exposed to 25°C drank the same amount of water but after 4 h of access to water, the heat‐acclimated rats had taken in 15.3 mL/kg compared to 11.1 mL/kg by the control rats (Fig. 3, top). Heat exposure caused an increase in water intake in both control and heat‐acclimated rats with the control rats drinking more than the heat‐acclimated rats. Three‐way ANOVA with repeated measures of the water intake data showed significant main effects of group (*F*_1,31_ = 12.46, *P* < 0.001), temperature (*F*_1,31_ = 95.08, *P* < 0.001), and time (*F*_4, 124_ = 112.71, *P* < 0.001) and significant interactions between group and temperature (*F*_1,31_ = 23.01, *P* < 0.001), temperature and time (*F*_4, 124_ = 7.46, *P* < 0.001), and group and time (*F*_4, 124_ = 2.61, *P* < 0.05), but no significant interaction among group, temperature, and time.

Prior heat exposure decreased urine output in both the control rats and the heat‐acclimated rats during the drink period (Fig. 3, middle). Three‐way ANOVA with repeated measures of the drink period urine output data showed significant main effects of group (*F*_1,31_ = 5.25, *P* < 0.05), temperature (*F*_1,31_ = 14.40, *P* < 0.001), and time (*F*_4, 124_ = 17.62, *P* < 0.001) and a significant interaction between group and temperature (*F*_1,31_ = 7.05, *P* < 0.02) but no other significant interactions.

Although the rats had free access to water for 4 h, they did not completely rehydrate (Fig. 3, bottom). The heat‐acclimated rats exposed to 25°C rehydrated to 83% but the remaining groups only rehydrated to the 50% level. The heat‐acclimated rats exposed to 37.5°C initially rehydrated more slowly than did the other three groups. Three‐way ANOVA with repeated measures of the percent rehydration data showed a significant main effect of time (*F*_4, 124_ = 65.80, *P* < 0.0001) but no significant main effect of either group or temperature. ANOVA also showed significant interactions between temperature and time (*F*_4, 124_ = 3.71, *P* < 0.01) and group and time (*F*_4, 124_ = 9.67, *P* < 0.05) but no significant interaction between group and temperature nor among group, temperature, and time.

Urine electrolyte concentrations are shown in Table 1. Two‐way ANOVA of the urine electrolyte concentrations during the exposure period showed that there were significant main effects of group on sodium (*F*_1,31_ = 4.32, *P* < 0.05) and potassium (*F*_1,31_ = 13.09, *P* < 0.001) concentrations, but no significant effects of temperature nor significant interactions between group and temperature. Two‐way ANOVA of the urine sodium concentration during the drink period data showed that there was a significant main effect of temperature (*F*_1,22_ = 8.87, *P* < 0.01) and a significant interaction between group and temperature (*F*_1,22_ =  6.09, *P* < 0.05) but no significant main effect of group. Two‐way ANOVA of the urine potassium concentration during the drink period data showed that there was a significant main effect of group (*F*_1,22_ = 15.73, *P* < 0.001) but no significant main effect of temperature nor significant interaction between group and temperature.

**Table 1:** Urinary electrolyte concentrations during and following thermal dehydration

Group	Exposure Na^+^ concentration (mmol/L)	Exposure K^+^ concentration (mmol/L)	Drink Na^+^ concentration (mmol/L)	Drink K^+^ concentration (mmol/L)
Con‐25°C	99 ± 19	174 ± 22	94 ± 11	169 ± 30
HA‐25°C	165 ± 19	263 ± 12	202 ± 30	303 ± 9
Con‐37.5°C	135 ± 21	156 ± 23	237 ± 34	203 ± 38
HA‐37.5°C	149 ± 17	207 ± 16	215 ± 28	297 ± 11

Effects of heat acclimation and heat exposure on mean ± SE urinary sodium and potassium concentrations during the exposure and drink periods. Con, Control rats; HA, Heat‐Acclimated rats. ANOVA indicated significant effects of group on sodium and potassium concentrations during the exposure period. ANOVA indicated a significant effect of temperature and a significant interaction between group and temperature on sodium concentration during the drink period and a significant effect of group on potassium concentration during the drink period.

### Effects of heat acclimation on blood indicators of body water status following thermal dehydration

Blood measures of body water status from control and heat‐acclimated rats following the exposure period are shown in Table 2. The heat‐acclimated group had lower hematocrit and plasma protein concentration at 25°C without showing differences in plasma osmolality or sodium or potassium concentration. Heat exposure increased plasma osmolality and plasma sodium concentration and decreased plasma potassium concentration in both groups and increased hematocrit in the heat‐acclimated group. Two‐way ANOVA indicated significant effects of group on hematocrit (*F*_1,30_ = 29.13, *P* < 0.01) and plasma protein concentration (*F*_1,30_ = 5.73, *P* < 0.05) but not on plasma osmolality, plasma sodium concentration, or plasma potassium concentration. Two‐way ANOVA indicated significant effects of temperature on hematocrit (*F*_1,30_ = 8.08, *P* < 0.01), osmolality (*F*_1,30_ = 13.79, *P* < 0.01), and plasma potassium concentration (*F*_1,30_ = 13.77, *P* < 0.01) but not on plasma protein concentration or plasma sodium concentration. There was a significant interaction between group and temperature only for hematocrit (*F*_1,30_ = 4.57, *P* < 0.05).

**Table 2:** Effect heat acclimation and heat exposure on blood indicators of body water status

Group	Hct	Plasma protein concentration (g/100 mL)	Plasma osmolality (mOs/kg)	Plasma Na^+^ concentration (mmol/L)	Plasma K^+^ concentration (mmol/L)
Con‐25°C	46.5 ± 0.4	6.8 ± 0.2	294 ± 2	145.0 ± 1.3	4.5 ± 0.1
HA‐25°C	40.2 ± 1.4	6.2 ± 0.2	295 ± 3	146.6 ± 2.0	4.4 ± 0.3
Con‐37.5°C	47.1 ± 0.5	6.5 ± 0.1	306 ± 2	148.8 ± 1.3	3.5 ± 0.2
HA‐37.5°C	44.4 ± 1.0	6.4 ± 0.1	300 ± 2	149.7 ± 2.9	3.7 ± 0.3

Effects of heat acclimation and heat exposure on mean ± SE hematocrit (Hct), plasma protein concentration, plasma osmolality, and plasma sodium and potassium concentrations. Con, Control rats; HA, Heat‐Acclimated rats. ANOVA indicated significant effects of group on hematocrit and plasma protein concentration and of temperature on hematocrit, osmolality and plasma protein concentration. ANOVA indicated a significant interaction between group and temperature on hematocrit.

### Thirst challenge experiments

Water deprivation *–* Table 3 shows intake and output data before and during the water deprivation period. The heat‐acclimated rats weighed significantly (*P* < 0.005) less than did the control rats. There was no significant difference in the amount of water the control and heat‐acclimated rats drank during the 1‐h pre deprivation period. However, the heat‐acclimated rats produced significantly (*P* < 0.005) less urine during this time than did the control rats. During the 24 h of water deprivation there were no significant differences in food intake or feces output between the two groups of rats, but the heat‐acclimated rats again produced significantly (*P* < 0.005) less urine. Although the heat‐acclimated rats lost a smaller proportion of their body weight during dehydration than did the control rats, this difference did not reach the level of statistical significance (*P* = 0.09).

**Table 3:** Effects of heat acclimation before and during water deprivation

Group	Initial BW (g)	During 1‐h pre deprivation		During 24 h of water deprivation			
		Water intake (mL/kg)	Urine output (mL/kg)	Food intake (g/kg)	Feces output (g/kg)	Urine output (mL/kg)	Change in BW (g/kg)
Con	361 ± 6	3.7 ± 0.6	3.0 ± 0.6	22.9 ± 1.3	11.6 ± 0.8	24.9 ± 0.7	58.0 ± 2.0
HA	299 ± 4^a^	4.4 ± 0.8	1.0 ± 0.3^a^	21.8 ± 1.4	9.6 ± 0.9	17.7 ± 0.8^a^	52.1 ± 2.6

Effects of heat acclimation on water intake and urine output for 1 h before water deprivation and on food intake, feces output, urine output and the change in body weight during 24 h of water deprivation at 25°C. Values are mean ± SE. BW, body weight; Con, Control rats; HA, Heat‐Acclimated rats.

a *P* < 0.005.

**Figure 4:**
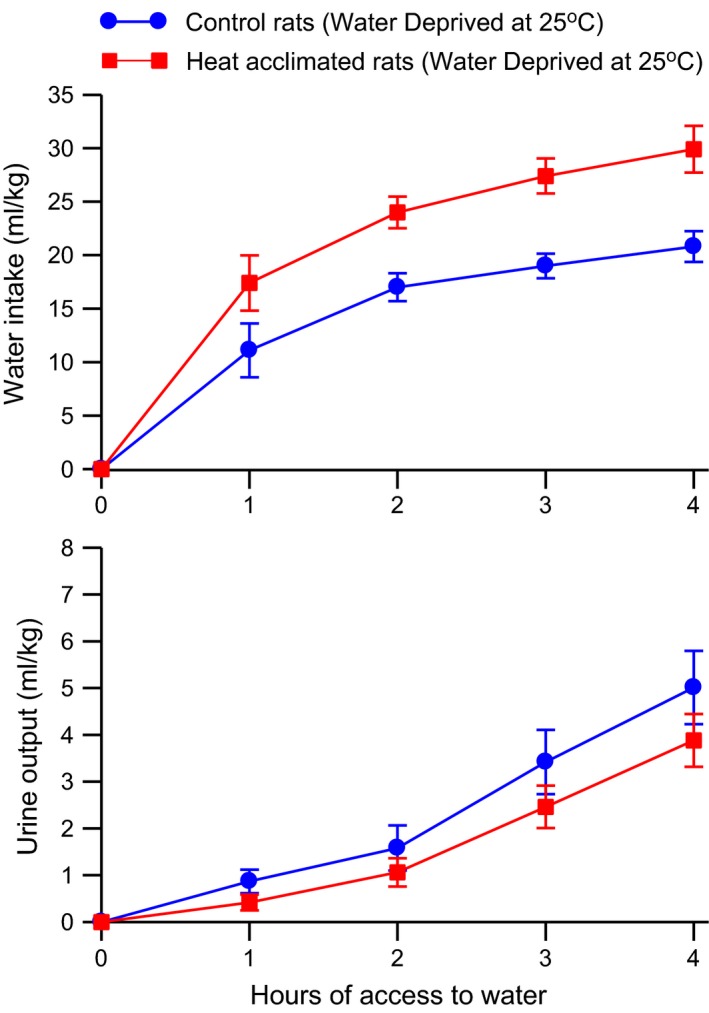
Mean ± SE cumulative water intake (*top*) and urine output (*bottom*) of control and heat‐acclimated rats for 4 h of access to water at an environmental temperature of 25°C following 24 h of water deprivation at an environmental temperature of 25°C. *n *=* *12 per group. ANOVA indicated significant effects of group and time on water intake and a significant effect of time on urine output.

Water intake and urine output of these rats during the drink period following 24 h of water deprivation are shown in Figure 4. Urine output was low in both groups with heat‐acclimated rats producing slightly less urine than the control rats. However, there was no statistical difference in the amount of urine produced between the two groups with two‐way ANOVA with repeated measures showing a significant (*F*_3,66_ = 49.52, *P* < 0.0001) effect of time but no significant main effect of group nor significant interaction between group and time. The heat‐acclimated rats drank more water than did the control rats. Two‐way ANOVA with repeated measures of the water intake data showed significant main effects of group (*F*_1,22_ = 12.64, *P* < 0.005) and time (*F*_3,66_ = 30.91, *P* < 0.0001) but no significant interaction between these two variables.

**Figure 5:**
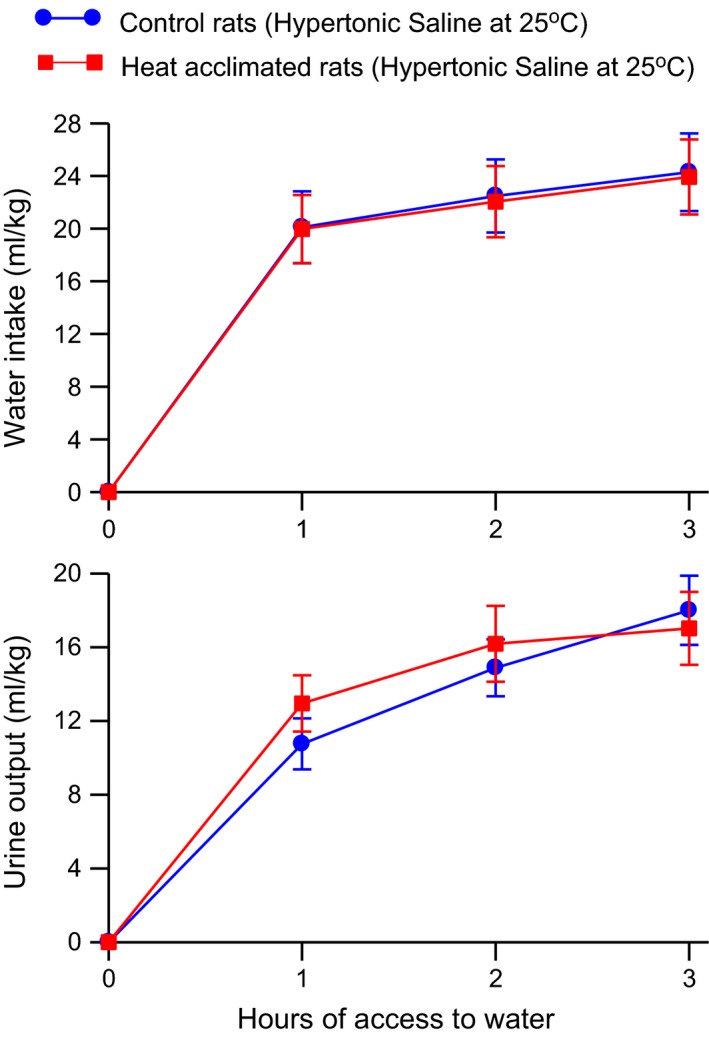
Mean ± SE cumulative water intake (*top*) and urine output (*bottom*) of control and heat‐acclimated rats for 3 h of access to water at an environmental temperature of 25°C following administration of 10 mL 0.75 mol/L NaCl/kg i.p. *n *=* *6 per group. ANOVA indicated only significant effects of time on water intake and urine output.

Hypertonic saline – Administration of hypertonic saline, which stimulates intracellular thirst, led to robust water intake and urine output in both groups of rats (Fig. 5). Heat acclimation did not alter either the water intake or urine output responses to injection of hypertonic saline. Two‐way ANOVA with repeated measures of the water intake data showed a significant effect of time (*F*_2,20_ = 64.51, *P* < 0.00001), but no significant effect of group nor significant interaction between group and time. Similarly Two‐way ANOVA with repeated measures of the urine output data showed a significant effect of time (*F*_2,20_ = 42.24, *P* < 0.00001), but no significant effect of group nor significant interaction between group and time. There was no significant effect of heat acclimation on sodium or potassium excretion rates following administration of hypertonic saline. Sodium excretion rates were 1317 *±* 127 *μ*mol/kg‐h for the control rats and 1352 *±* 73 *μ*mol/kg‐h for the heat‐acclimated rats. Potassium excretion rates were 600 *±* 60 *μ*mol/kg‐h for the control rats and 601 *±* 34 *μ*mol/kg‐h for the heat‐acclimated rats.

**Figure 6:**
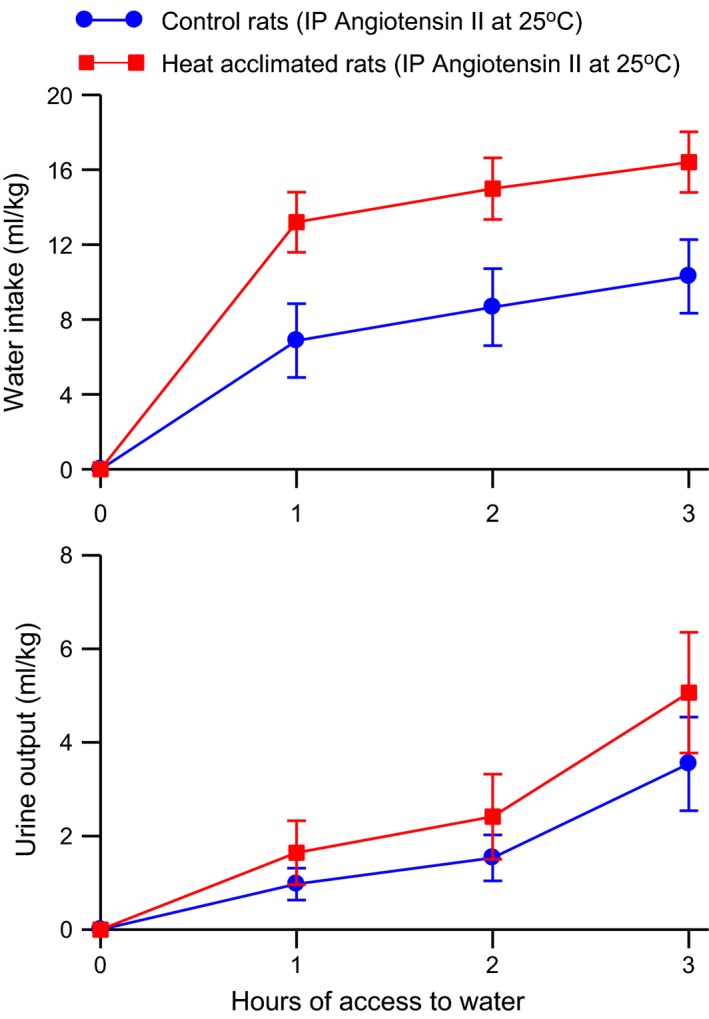
Mean ± SE cumulative water intake (*top*) and urine output (*bottom*) of control and heat‐acclimated rats for 3 h of access to water at an environmental temperature of 25°C following administration of 200 *μ*g angiotensin II/kg i.p. *n *=* *12 for the control group and 11 for the heat‐acclimated group. ANOVA indicated significant effects of group and time on water intake and a significant effect of time on urine output.

Intraperitoneal angiotensin II *–* Heat acclimation increased the drinking response to angiotensin II without altering the urine output response (Fig. 6). Two‐way ANOVA with repeated measures showed a significant effect of group (*F*_1,21_ = 5.62, *P* < 0.05) and a significant effect of time (*F*_2, 42_ = 156.7, *P* < 0.0001), but no significant interaction between group and time on water intake. There was only a significant effect of time (*F*_2, 42_ = 18.75, *P* < 0.0001) on urine output. In a similar experiment, there were no significant effects of heat acclimation on water intake or urine output responses to a control injection of 1 mL/kg 0.9% NaCl solution (2‐h water intake = 2.6 ± 0.8 mL/kg for control rats and 3.0 ± 0.7 for heat‐acclimated rats and 2‐h urine output = 1.6 ± 0.7 mL/kg for control rats and 1.2 ± 0.7 for heat‐acclimated rats).

**Figure 7:**
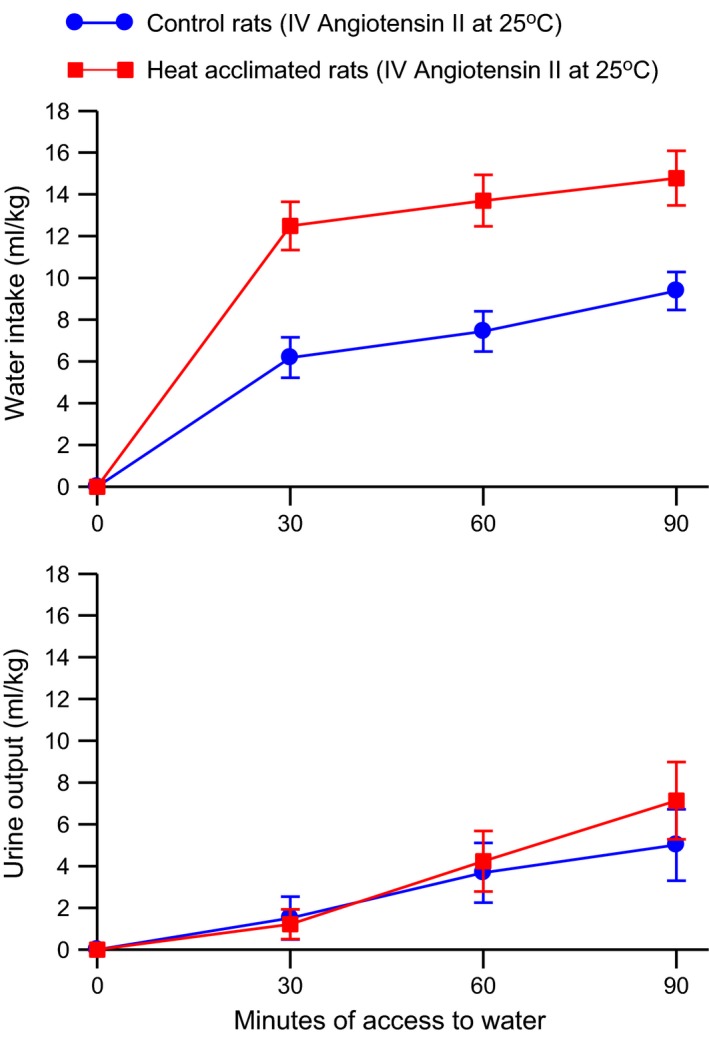
Mean ± SE cumulative water intake (*top*) and urine output (*bottom*) of control and heat‐acclimated rats for 90 min of access to water at an environmental temperature of 25°C and i.v. infusion of 0.25 *μ*g angiotensin II/kg‐min. *n *=* *8 per group. ANOVA indicated significant effects of group and time on water intake and a significant effect of time on urine output.

Intravenous angiotensin II – Heat‐acclimated rats drank more water than control rats during infusion of angiotensin II without showing any difference from control rats in urine output (Fig. 7). Two‐way ANOVA with repeated measures indicated a significant effect (*F*_1, 14_ = 16.21, *P* < 0.002) of group on water intake and a significant effect of time (*F*_2, 28_ = 24.49, *P* < 0.0001), but no significant interaction between group and time. There were no significant effects except for time (*F*_2, 16_ = 18.49, *P* < 0.0001) on urine output. Water intake and urine output were small during the 30‐min control infusion period and there were no significant differences between the control and heat‐acclimated rats (data not shown).

### Cardiovascular responses to angiotensin II

**Figure 8:**
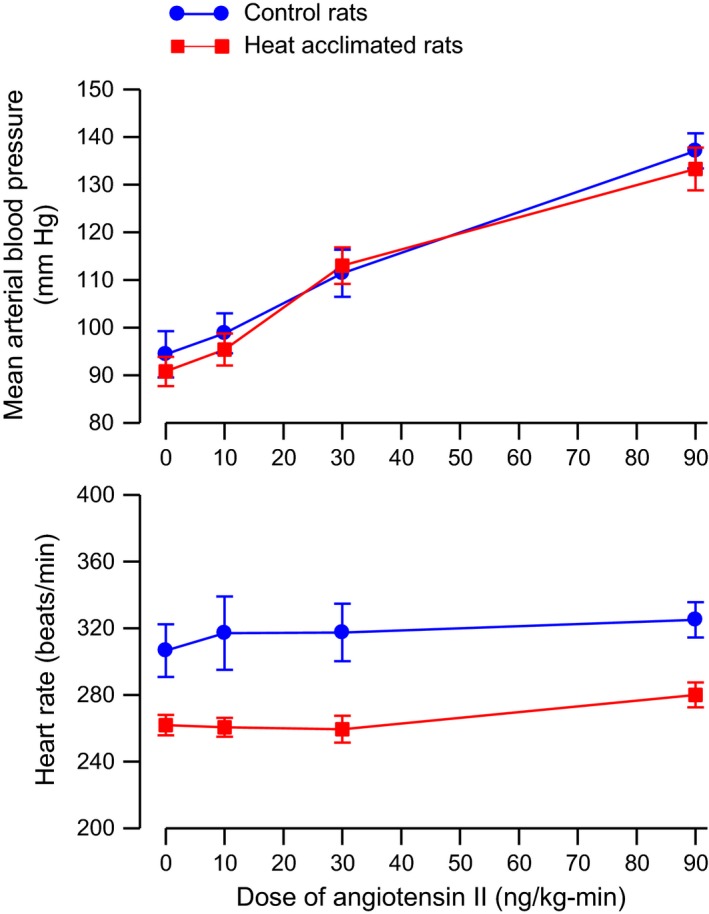
Mean ± SE mean arterial blood pressure (*top*) and heart rate (*bottom*) of anesthetized control and heat‐acclimated rats during i.v. infusion of various doses of angiotensin II. *n *=* *8 per group. ANOVA indicated significant effects of dose of angiotensin II on arterial blood pressure and significant effects of group and dose of angiotensin II on heart rate.

Angiotensin II increased mean arterial blood pressure in a dose‐dependent manner in both control and heat‐acclimated rats (Fig. 8, top). Heat acclimation did not alter the pressor response to angiotensin II. Two‐way ANOVA with repeated measures of the mean blood pressure data showed a significant main effect of dose of angiotensin (*F*_3,42_ = 97.74, *P* < 0.001), but no effect of group nor interaction between group and dose of angiotensin. The mean heart rates of the heat‐acclimated rats were less than those of the control rats at each dose of angiotensin used (Fig. 8, bottom). Angiotensin had minimal but significant effects on heart rate. Two‐way ANOVA with repeated measures of the heart rate data showed a significant main effects of group ((*F*_1,14_ = 9.57, *P* < 0.01) and dose of angiotensin (*F*_3,42_ = 3.21, *P* < 0.05) but no interaction between group and dose of angiotensin.

## Discussion

In rats, heat acclimation is associated with a wide range of gene expression, physiological, and behavioral changes, which improve tolerance to heat exposure (Horowitz et al. 1983, 1999; Gordon 1993; Schwimmer et al. 2004; Tetievsky et al. 2014). As thirst and subsequent water intake play important roles in reducing the deleterious impacts of heat exposure, we investigated the effects of heat acclimation on thirst in rats in this study.

As in other studies (Kotby and Johnson 1967; Elmér and Ohlin 1970; Kuroshima et al. 1982) heat acclimation reduced the growth rate and led to lower body weights in the current experiments. Additional physiological effects of heat acclimation include decreased daily food intake and increased daily water intake (Attah and Besch 1977; Gwosdow et al. 1985) and decreased oxygen consumption at environmental temperatures of 31–34°C (Kuroshima et al. 1982; Arieli and Chinet 1986; Shido and Nagasaka 1990). Heat acclimation has been reported to decrease (Gwosdow et al. 1985), increase (Horowitz and Samueloff 1979), and not change total body water (Attah and Besch 1977). Rats demonstrate an initial increase in activity during heat exposure, presumably as an escape reaction, but then reduce activity as heat exposure continues (Lewis et al. 1960).

During acute exposure to heat without access to water, both control and heat‐acclimated rats became thermally dehydrated due to significant increases in evaporative and urinary water losses. Acute heat exposure also caused an increase in feces output as previously reported (Barney et al. 1995). The increased feces output is a stress response (Bonaz and Taché 1993; Morrow and Garrick 1997; Banji et al. 2014) and thus the reduced feces output in the heat‐acclimated rats is another indication that they are less stressed by acute heat exposure.

Heat acclimation significantly reduced the level of thermal dehydration with smaller water losses than in control rats, even though heat acclimation increases the relative weight of the submaxillary gland (Elmér and Ohlin 1970; Horowitz 1976), which would allow for increased salivation. Heat acclimation in rats has been reported to decrease (Horowitz et al. 1983; Schwimmer et al. 2004) and not change (Horowitz et al. 1999) the colonic threshold temperature at which saliva production increases. Heat acclimation shortens the latency to initiate salvia spreading (Horowitz et al. 1983), but reduces salivation during the plateau phase of hyperthermia during heat stress (Horowitz et al. 1983). During this time, colonic temperature in heat‐acclimated rats is also reduced (Horowitz et al. 1983; Shochina and Horowitz 1989; Epstein et al. 1990) although this effect was not observed when using a radiotelemetry method of measuring core temperature, where a reduction in core temperature in heat‐acclimated rats was only observed during the initial stage of heat exposure (Matthew 1997). Because core temperature does not directly alter water intake in thermally dehydrated rats (Barney and Folkerts 1995), differences in core temperature between control and heat‐acclimated rats are probably not responsible for differences in water intake. Heat acclimation reduces heat production during heat exposure (Kuroshima et al. 1982; Arieli and Chinet 1986; Shido and Nagasaka 1990) and increases dry heat loss (Shochina and Horowitz 1989; Shido and Nagasaka 1990; Horowitz et al. 1999), which allow the heat‐acclimated rats to reduce evaporative water loss during heat exposure while still maintaining core temperature at tolerable levels.

At 25°C both plasma protein concentration and hematocrit were lower in the heat‐acclimated rats than in the control rats, suggesting that heat acclimation led to an increase in plasma volume, which agrees with some (Kuroshima et al. 1982; Horowitz and Givol 1989), but not other (Horowitz 1976; Horowitz and Samueloff 1979; Gwosdow et al. 1985; Horowitz et al. 1999) studies on rats. Differences in blood sampling technique or the environmental temperature at which the blood samples were taken may explain these differences. Heat acclimation did not cause significant changes in plasma osmolality, similar to other studies (Epstein et al. 1990; Horowitz et al. 1999), or plasma sodium or potassium concentrations when measured at 25°C, indicating that heat‐acclimated rats do not shift osmotic balance.

Heat‐acclimated rats had a small increase in water intake following 4 h of exposure to 25°C compared to the control rats. Thermal dehydration led to significant increases in water intake in both control and heat‐acclimated rats, with heat‐acclimated rats having lower water intakes than control rats (39% less) following 4 h of heat exposure without access to water. The reduced water losses of the heat‐acclimated rats during thermal dehydration led to smaller increases in plasma osmolality and plasma sodium concentration, confirming previous results (Epstein et al. 1990; Horowitz et al. 1999). As thermal dehydration‐induced thirst is primarily intracellular in nature (Nose et al. 1985, 1986; Barney and West 1990; Barney 1997), the reduction in the stimuli for intracellular thirst in the heat‐acclimated rats led to a decrease in water intake. In addition to having lower total water intakes, the heat‐acclimated rats drank more slowly after heat exposure than did the control rats. After 1 h of access to water, control rats had ingested 77.2% of their total 4‐h water intake, whereas the heat‐acclimated rats had only ingested 57.3% of their total 4‐h water intake. The lower initial rate of drinking and rehydration of the heat‐acclimated rats may indicate that the initial termination of thermal dehydration‐induced drinking, which probably depends on presystemic signals related to gastrointestinal distension and osmolality/sodium concentration (Blass and Hall 1976; Stricker and Hoffmann 2007), is enhanced by heat acclimation.

Although both control and heat‐acclimated rats had free access to water, they did not rehydrate completely following acute thermal dehydration. By the end of the 4‐h drink period both the thermally dehydrated control and heat‐acclimated rats had rehydrated approximately 50%, although the control rats had higher levels of rehydration during the first hour of access to water. The lack of full hydration of the thermally dehydrated rats agrees with earlier studies (Nose et al. 1985, 1986; Barney and West 1990; Barney and Folkerts 1995; Barney et al. 1995, 1999) and has been attributed to the removal of the osmotic drive to drink by returning plasma osmolality and sodium concentration to control levels prior to ingestion of water equal to the water losses (Nose et al. 1985, 1986; Barney and West 1990; Barney 1997). This reflects, in part, the fact that during thermal dehydration there is a loss of sodium and other electrolytes in the urine and saliva. Because of the similar levels of rehydration in the thermally dehydrated control and heat‐acclimated rats after 4 h of access to water, it does not appear that heat acclimation alters the primary stimulus for thermal dehydration‐induced thirst.

Heat‐acclimated rats retained more of the water they drank than did the control rats after 4 h of exposure to 25°C, but not after 4 h of exposure to 37.5°C. In addition, heat‐acclimated rats generally had higher urinary sodium and potassium concentrations than the control rats. These differences may indicate that heat acclimation brings about alterations in renal function in rats. In an earlier study, heat‐acclimated rats had lower rates of urine production as well as reduced clearances of inulin, urea, and p‐aminohippurate compared to control rats (Chayoth et al. 1984), suggesting that heat acclimation reduces renal blood flow and glomerular filtration rate. In another study (Horowitz et al. 1985), however, heat acclimation increased renal blood flow. Heat acclimation did not alter basal plasma arginine vasopressin (AVP) levels, but enhanced the AVP response to thermal dehydration (Epstein et al. 1990). This AVP response does not prevent the increase in urine output that is observed during the first hour of heat exposure (Barney and West 1990), but does lead to minimal urine output during the drink period.

In order to gain a better understanding of the effects of heat acclimation on thirst in rats, we also determined water intake in rats in which thirst was induced by means other than thermal dehydration. Heat‐acclimated rats drank significantly more water following 24 h of dehydration at 25°C than did control rats. The increased water intake of the heat‐acclimated rats was not due to a greater level of dehydration, since the heat‐acclimated rats drank the same amount of water in the 1‐h adjustment period prior to water deprivation, had lower urine volumes during that time and during the 24 h of water deprivation, ate the same amount of food, and lost slightly less relative body weight than did the control rats. As the heat‐acclimated rats had the same low urine outputs during the drink period as did the control rats, they were more successful at rehydration following water deprivation than the control rats. Water deprivation is associated with an increase in urinary sodium excretion in rats (McKinley et al. 1983) which acts to limit rehydration (Blass and Hall 1976), but it remains unlikely that the lower water intake in control rats following water deprivation is due to lower plasma sodium levels as heat acclimation significantly increased urinary sodium concentration without significantly changing urine volume. As thirst due to water deprivation has both an intracellular and a volemic/angiotensin II component (Stricker and Sved 2000; McKinley et al. 2004) we determined the effects of these thirst stimuli individually in heat‐acclimated rats.

Administration of intraperitoneal hypertonic saline led to vigorous and equal drinking in control and heat‐acclimated rats. The hypertonic saline load and the drinking led to similar increases in urine output and sodium and potassium excretion rates in both groups. Thus, as the heat‐acclimated and control rats drank the same amount of water in response to the same osmotic challenge, it appears that heat acclimation does not alter the intracellular thirst response. However, experiments in which the drinking response of control and heat‐acclimated rats to various doses of hypertonic saline might demonstrate some differences in sensitivity to osmotic challenge.

Heat‐acclimated rats drank significantly more water than control rats in response to either intraperitoneal injection or intravenous infusion of angiotensin II, which is one mediator of volemic thirst (McKinley et al. 2004; Johnson 2007; Thornton 2010) while having the same urine output as the control rats. Thus, heat acclimation appears to increase the sensitivity to the potent dipsogenic hormone, angiotensin II. Heat acclimation in rats caused an increase in the hypothalamic cell membrane to cytosol ratio of both AT_1_ and AT_2_ receptors (Schwimmer et al. 2004) and an AT_1_ receptor agonist and an AT_2_ receptor antagonist had greater effects on thermoregulatory functions in heat‐acclimated rats than in control rats (Horowitz et al. 1999; Schwimmer et al. 2004). An increased drinking response to angiotensin II may also explain the increased water intake in the heat‐acclimated rats following water deprivation. However, the regulation of water intake is complex and involves both peripheral and central components and thirst satiation involves both presystemic and systemic factors (Stricker and Sved 2000; McKinley et al. 2004; Johnson 2007; Stricker and Hoffmann 2007; Krause et al. 2010; Thornton 2010). Determination of the role of these factors was beyond the scope of this study.

To determine if heat acclimation altered nonthirst responses to angiotensin II, we determined the pressor responses of anesthetized control and heat‐acclimated rats to infusion of angiotensin II. Initial heart rate was significantly lower in the heat‐acclimated rats, which has been observed in some (Horowitz and Meiri 1993; Matthew 1997) but not other (Horowitz et al. 1985; Moran et al. 1996) studies. As in other studies (Moran et al. 1996; Matthew 1997) heat acclimation did not alter resting arterial blood pressure. Infusion of angiotensin II caused a dose‐dependent increase in mean arterial blood pressure that was the same in control and heat‐acclimated rats. Thus, heat acclimation does not appear to alter all responses to angiotensin II, but its specificity on thirst responses remains to be fully determined. Heat acclimation has been reported to not alter the contractile effects of norepinephrine on mesenteric, caudal, or femoral arteries (Looft‐Wilson et al. 1999; Li et al. 2008) but to increase the contractile response of aortic rings to norepinephrine and phenylephrine (Shochina et al. 1996; Li et al. 2008). Heat acclimation lead to an increased relaxation response of mesenteric artery rings to pilocarpine (Haddad and Horowitz 1999) and to decreased relaxation responses of both aortic and portal vessel rings to isoproterenol (Shochina et al. 1996). Such changes are suggested to play a role in changing blood flow in various regions in heat‐acclimated rats.

## Perspectives

Heat acclimation in rats leads to a suite of physiological changes that allow the rats to maintain homeostasis in a hot environment in a more economical manner. For example, reductions in heat production and increases in dry heat loss allow for maintenance of body temperature with less loss of water for evaporative cooling. We found that heat acclimation enhanced the water intake response to angiotensin II, a hormone that plays an important role in extracellular/volemic thirst, without altering the water intake responses to increases in plasma osmolality/sodium concentration induced by administration of hypertonic saline. Furthermore, heat acclimation increased the water intake and improved hydration after 24 h of water deprivation. On other hand, heat acclimation did not alter the ability of rats to rehydrate following thermal dehydration, but did reduce the rate at which thermal dehydration developed. Heat acclimation in rats does not appear to improve water balance following heat exposure through enhanced drinking, but does reduce the amount of water needed to maintain homeostasis. Additional studies on the effects of heat acclimation on the pathways of water balance and studies on possible alterations in specific thirst pathways following heat acclimation in humans may prove worthwhile. A thorough understanding of biological responses to sustained heat exposure is of critical importance as global warming continues.

## Conflict of Interest

None declared.
